# Mathematical analysis of fractional Chlamydia pandemic model

**DOI:** 10.1038/s41598-024-82428-1

**Published:** 2024-12-28

**Authors:** Zuhur Alqahtani, Areej Almuneef, Mahmoud H. DarAssi, Yousef AbuHour, Mo’tassem Al-arydah, Mohammad A. Safi, Bashir Al-Hdaibat

**Affiliations:** 1 Department of Mathematical Science, College of Science, Princess Nourah bint Abdulrahman University, P.O.Box 84428, 11671 Riyadh, Saudi Arabia; 2 Department of Basic Sciences, Princess Sumaya University for Technology, P.O.Box 11941, Amman, Jordan; 3 Department of Mathematics, Khalifa University, Abu Dhabi, UAE; 4 Department of Mathematics, Faculty of Science, The Hashemite University, P.O.Box 330127, Zarqa, 13133 Jordan

**Keywords:** Stability analysis, Fractional derivatives, Sensitivity analysis, Equiliburia, Applied mathematics, Computational models, Infectious diseases

## Abstract

In this study, we developed a Caputo-Fractional Chlamydia pandemic model to describe the disease’s spread. We demonstrated the model’s positivity and boundedness, ensuring biological relevance. The existence and uniqueness of the model’s solution were established, and we investigated the stability of the $\alpha$-fractional order model. Our analysis proved that the disease-free equilibrium point is locally asymptotically stable. Additionally, we showed that the model has a single endemic equilibrium point, which is globally asymptotically stable when ${\mathscr {R}}_0$ exceeds 1. Using Latin Hypercube sampling and partial rank correlation coefficients (PRCCs), sensitivity analysis identified key parameters influencing ${\mathscr {R}}_0$. Numerical simulations further illustrated the impact of parameter variations on disease dynamics.

## Introduction

The world is witnessing a notable shift towards mathematical modeling as a critical instrument for understanding infectious diseases, providing vital knowledge for forecasting and controlling future outbreaks. In this scenario, we concentrate on a mathematical model designed to understand the dynamic transmissions of Chlamydia, a widespread public health concern, particularly in the United States. Chlamydia despite often being asymptomatic, Chlamydia ranks among the bacterial infections that are reported most frequently, with alarming estimates indicating millions of new cases in 2018 alone, primarily affecting young people aged 15–24. Approximately one in twenty sexually active young women aged 14–24 are estimated to have Chlamydia infection^1^. If Chlamydia infections are left untreated, they can lead to serious health problems, Notably inflammatory disease of the pelvis (PID), which can lead to complications like tubal blockage, pregnancy outside the uterus, and persistent pelvic and abdominal pain.

Differential equations are central in the mathematical modeling of infectious diseases within epidemiology^2–6^. Differential equations describe changes in disease spread dynamics. One of the most famous compartmental models, SIR-Susceptible, Infectious, Recovered-is based on ordinary differential equations calculating the number of infected people. Over the last few years, fractional differential equations have become prominent in describing complicated epidemic dynamics. Researchers work on the stability, equilibria, and bifurcations of disease models, applying numerical methods to simulate the progress of diseases. Inevitably, such models provide insight into health policy, vaccination strategy, and the control of outbreaks. For example, during the COVID-19 pandemic, some mathematical models calculated the extent of infections, measured the impact of interventions such as lockdown and vaccination campaigns, and recommended decisions. These models study variables, including transmission rates, incubation periods, and population demographics^7–10^.

Even though there was a temporary decrease in reported Chlamydia cases in 2020 due to COVID-19 restrictions, it is necessary to increase the research efforts aimed at understanding this disease and reducing its impact on public health. Mathematical models are essential in investigating this disease’s spread by better understanding the disease dynamics and guiding effective control measures. The importance of integrating memory effects into these models is noteworthy. Fractional calculus, an emerging field, shows potential in developing models that properly consider memory effects, improving the accuracy of disease predictions, and empowering control strategies. Our efforts in research are inspired by the high drive to look deeper and tune these models to face off much better the tests that arise around Chlamydia and other infectious diseases. Apart from the fractional calculus-based models, the integer models are developed with utmost precision to handle the dynamics of certain infections such as Chlamydia trachomatis and Gonorrhea to a possible extent co-infections. Such models reveal the interaction of various pathogenic agents and increase our understanding of transmission dynamics. The following references provide more extensive coverage of these integer models : This has led to the rigorous definition of different types of fractional derivatives, which are widely used today because of their intricate properties, such that through a closer look, one could more genuinely study the phenomena exposed in reality. Applications of this new and promising field have been made with quite some success. Several studies also confirmed that $alpha-$fractional derivatives are very effective in modeling and analyzing complex problems. For example, with the base data from real data, Qureshi et al.^11^ adopted a fractional order model mimicking the dynamics of blood ethanol concentration in a mathematical way. Baleanu et al.^12^ studied the cholera epidemic dynamics by fitting an $\alpha -$fractional order model with realistic data. Vellappandi et al.^13^ engaged both classical and corresponding fractional derivative operators for the dynamic transmissions of the COVID epidemic. Kumar et al.^14^ have described a nonlinear mathematical structure that involves integer and fractional derivatives for studying the transmission dynamics of dental caries in humans. On a different note, Vellappandi et al.^15^ developed some optimal control strategies with the Caputo fractional derivative for handling mosaic epidemics. Erturk et al.^16^ conducted a special study with the help of Caputo fractional derivatives to model corneal shape. Kumar et al. derived a model for the mechanism of the alkali-silica chemical reaction within the Caputo context for nonlinear formulations. Furthermore, Rezapour et al.^17^ analyzed a 3D Hopfield neural network model by incorporating Atangana-Baleanu operators, illustrating the adaptability of fractional calculus in neural network models. Abbas et al.^7^ introduced innovative stability and bifurcation analyses for an HIV-1 mathematical model with a discrete-time delay, highlighting the usefulness of fractional derivatives in understanding the dynamics of infectious diseases. These varied studies emphasize the extensive applications and growing interest in using fractional derivatives to analyze and understand complex real-world situations, providing promising directions for future research and practical application.

The motivation for developing a fractional model lies in its ability to capture memory effects and hereditary properties that traditional integer-order models cannot. Fractional calculus provides a more comprehensive framework for modeling complex dynamical systems where past states influence current behavior, making it particularly useful in various fields. For instance, fractional calculus was applied to model electrically stimulated muscle dynamics in the gazelle optimization expedition^18^, showcasing its potential in biomedical engineering where systems exhibit time-dependent behavior. In another study, a fractional-order model for Parkinson’s disease was developed to simulate brain electrical activity rhythms better, offering a more accurate representation of the disease’s progression and aiding in developing more effective treatments. Additionally, fractional calculus has been applied in designing advanced algorithms like fractional hierarchical gradient descent and normalized fractional gradient methods, which improve parameter estimation and control in nonlinear systems. These examples underscore the value of fractional calculus in enhancing model accuracy and providing deeper insights into system behavior, justifying the development of fractional models for diverse applications, including disease transmission modeling.

Future research on Chlamydia transmission using fractional order models can benefit from recent advancements in neural network algorithms. Studies such as those by^19,20^, and^21^ have demonstrated the potential of integrating fractional calculus with neural networks to enhance the accuracy and efficiency of disease spread models. Developing hybrid models that combine supervised, unsupervised, and reinforcement learning techniques, as discussed in these studies, could lead to more robust and adaptable algorithms for predicting Chlamydia dynamics. As suggested by these works, exploring the impact of memory effects and long-term dependencies in fractional order models can further improve understanding of disease transmission patterns. These future research directions aim to refine existing methodologies and open new avenues for applying fractional order models in epidemiology, ultimately contributing to more effective disease control and prevention strategies.

The proposed model is compared with existing models based on several key features:

- Number of Compartments: Including additional compartments in epidemiological models enhances the understanding and prediction of disease spread by providing a more detailed representation of the population dynamics.
- Memory Effects: Utilizing fractional derivatives allows the model to account for the entire process history, capturing long-term dependencies crucial in epidemiology, where past states significantly influence current disease spread.
- Sensitivity Analysis of $R_0$: This involves analyzing how variations in model parameters impact the basic reproduction number $R_0$, which is essential for understanding the potential for disease outbreaks and the effectiveness of control measures.
- Vaccination: Incorporating vaccination into the model reduces the number of susceptible individuals, thereby decreasing the overall pool of individuals who can contract and spread Chlamydia, which is critical for effective disease control.
- Prediction Accuracy: Accurate predictions in each compartment are vital for understanding the disease dynamics and implementing effective control measures, ensuring timely and appropriately targeted interventions.
- Stages of the Developmental Cycle and Density of Infected Cells in Stage: This feature involves tracking the number of cells infected with Chlamydia at various stages of its biphasic life cycle, providing valuable insights into disease progression and the efficacy of treatments.

The Caputo definition of fractional derivative is often preferred over other definitions in epidemiological modeling due to its compatibility with traditional initial conditions, typically in terms of integer-order derivatives. This makes it easier to incorporate real-world data into the models. Additionally, the Caputo derivative provides a more intuitive physical interpretation, especially in biological systems, by representing memory effects where past states influence present and future dynamics. This is crucial in epidemiology, where the history of disease spread impacts current transmission rates. Furthermore, the Caputo derivative offers greater flexibility in modeling complex systems, capturing non-local behavior and memory effects more accurately than integer-order models. It also ensures a smooth transition from fractional-order to integer-order models, facilitating comparisons and transitions between models.

In this paper, we mainly consider the Caputo fractional-order Chlamydia pandemic model. The fractional derivatives are defined, and their properties are listed in section “Preliminaries”. The epidemiology model is formulated and analyzed in section “Mathematical model formulation and design”. In sections “Equilibrium stability analysis” and “Endemic equilibrium”, we have conducted the analysis of stability for the proposed model. $R_0$ is computed, and local and global stability analyses are performed. In section “Discussion and conclusion”, we introduce the sensitivity analysis, and the results are illustrated by numerical simulation. The conclusion is presented in section “Conclusion”.

## Preliminaries

### Definition 1

^22^ For $t>0$, the gamma function is defined as follows:

$$\begin{aligned}\Gamma (t)=\int _0^{\infty } y^{t-1}\,e^{-y}\,dy\end{aligned}$$

### Definition 2

^22^ The Rieman–Liouville fractional derivative of order $\alpha \in [n-1,n)$ of the function *f* is defined by

$$\begin{aligned}^{RL}D^{\alpha }f(x)=\frac{1}{\Gamma (n-\alpha )}\frac{d^n}{dx^n}\int _0^x(x-t)^{n-\alpha -1}\,f(t)dt, \quad n=[\alpha ]+1\end{aligned}$$

### Definition 3

^22^ The Caputo-fractional derivative of fractional $\alpha \in (n-1,n]$ for an $n^{th}$-differentiable function *f*(*y*) is given by the following integral formula:

$$\begin{aligned}^CD^{\alpha }f(y)=\frac{1}{\Gamma (n-\alpha )}\int _0^y(y-t)^{n-\alpha -1}\,f^{(n)}(t)dt, \quad n=[\alpha ]+1\end{aligned}$$

### Definition 4

^22^(Linear property of fractional derivatives) For the continuous functions *f*, *g* and the scalars $k_1$ and $k_2$, we have

- $$^{RL}D^{\alpha }(k_1\,f(y)+k_2\, g(y))=k_1\,^{RL}D^{\alpha }f(y)+k_2\,^{RL}D^{\alpha }g(y)$$
- $$^{C}D^{\alpha }(k_1\,f(y)+k_2\, g(y))=k_1\,^{C}D^{\alpha }f(y)+k_2\,^{C}D^{\alpha }g(y)$$

### Theorem 1

^23^ *Any contractive operator* $T: X\longrightarrow X$ *that maps a complete metric space onto itself has only one fixed point* $T(x^*)=x^*$. *Moreover, T satisfies the condition below*

$$\begin{aligned}dist(T(x^*),T(y)))< L\,dist(x^*,y), \quad 0<L<1.\end{aligned}$$

### Definition 5

^24^ For integrable function $f:{\mathbb {R}}\longrightarrow {\mathbb {R}}$ and $0<\alpha \le 1$, the fractional-integral for the function *f* of order $\alpha$ is given by

$$\begin{aligned}I^{\alpha }f(y)=\frac{1}{\Gamma (\alpha )} \int _0^y (y-t)^{\alpha -1}f(t) dt\end{aligned}$$

### Lemma 1

^25^ Representing the Laplace transform associated with the Caputo fractional derivative:

$$\begin{aligned} {\mathscr {L}}\{ ^CD^{\alpha } f(t)\}=s^{\alpha }\, {\mathscr {L}}\{ f(t)\}-s^{\alpha -1}\,f(0), \quad 0<\alpha <1 \end{aligned}$$

### Definition 6

^22^ Let $\alpha>0,\,\beta >0$ and ${\mathbb {C}}$ be the complex plane. If $z\in {\mathbb {C}}$, then the two parameters Mittag-Leffler function is given by

$$\begin{aligned}E_{\alpha ,\beta }(z)=\sum _{n=0}^{\infty } \frac{z^n}{\Gamma (n\alpha +\beta )} \end{aligned}$$

### Theorem 2

^26^ *The equilibrium solutions* $x^*$ *of the Caputo-fractional differential equations system*

$$\begin{aligned}^CD^{\alpha }x(t)=f(t,x), \quad x(t_0)=x_0\end{aligned}$$

*The Jacobian matrix* $\dfrac{\partial f}{\partial x_j}$ *evaluated at equilibrium points ensures local asymptotic stability (LAS) if its eigenvalues* $\lambda _j$ *satisfy*

$$\begin{aligned}\left| \arg (\lambda _j)\right| =\pi >{\alpha \pi }/{2},\quad 0<\alpha <1,\quad \forall j=1,\cdots , n.\end{aligned}$$

### Theorem 3

^27^ *Let* $x(t) \in {\mathbb {R}}^+$ *be a continuous and derivable function. If* $x^* \in {\mathbb {R}}^+$ *and* $0<\alpha <1$, *then for any time instant* $t\ge t_0$, *we have*

$$\begin{aligned}^CD^{\alpha }\,\left[ x(t)-x^*-x^*\,\ln {\frac{x(t)}{x^*}}\right] \le \left( 1-\frac{x(t)}{x^*}\right) \ ^CD^{\alpha } x(t). \end{aligned}$$

### Theorem 4

^28^ *Let* $x^*$ *the equilibrium point of* $^CD^{\alpha } x(t) = f (t,x)$, *where* $\Omega \in {\mathbb {R}}^n$ *be a domain containing* $x^*.$ *By letting* $V(t,x): {\mathbb {R}}^+\bigcup \{0\} \times \Omega \longrightarrow {\mathbb {R}}$, *and V is a continuously differentiable (Lyapunov candidate) map such that*

- $$W_1(x) \le V(t,x) \le W_2(x)$$,
- $- ^CD^{\alpha } V(t,x) \ge -W_3(x)$,  * for all nonnegativet and for all* $x\in \Omega , \alpha \in (0,\,1).$

*for given continuous positive definite functions* $W_1(x),W_2(x)$ *and* $W_3(x)$ *on* $\Omega$. *Then* $x^*$ *is globally asymptotically stable.*

## Mathematical model formulation and design

In this section, we proposed a dynamical fractional-derivative Chlamydia mathematical model deduced from the model presented by Sharomi and Gumel^29^.

**Fig. 1:**
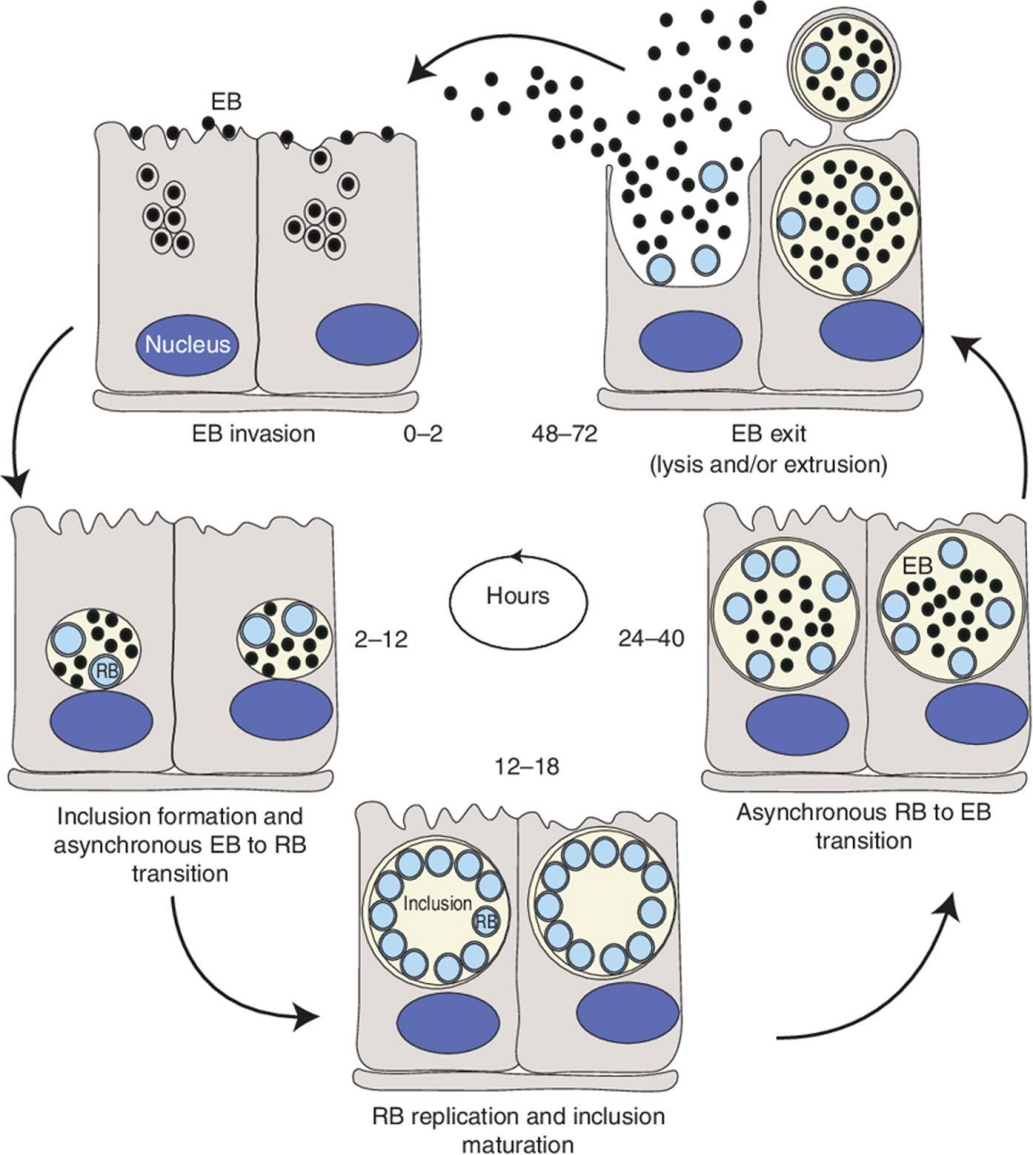
The life cycle of Chlamydia^30^.

The life cycle of Chlamydia involves elementary bodies (EBs) forming a nascent inclusion within 2 h of cell entry, differentiating into reticulate bodies (RBs) between 2 and 6 h, with RBs dividing by binary fission by 12 h, peaking in numbers by 18–24 h, and then differentiating back to EBs around 24 h, continuing until lysis or release occurs between 48 and 72 h, depending on the species. Figure 1 depicts the life cycle of Chlamydia.

The proposed model comprises eight coupled ODEs and eight compartments: The density of healthy epithelial cells within the host at a given time $t$ is $H$. The density of *Chlamydia* in its elementary body (EB) morphological form at time $t$ is represented by $E(t)$. The density of *Chlamydia* in its reticulate body (RB) morphological form at time $t$ is represented by $R(t)$. An elementary body (EB) infects a healthy epithelial cell and undergoes *five* developmental stages at the beginning of the EBs are released to propagate the cycle. This process is presented as follows^31^:

- Phase 1: The EB is attached and included to the surface of the epithelial cell in the host.
- Phase 2: The EB is converted to RB forms inside the infected epithelial cell.
- Phase 3: The RB multiplies inside the infected epithelial cell.
- Phase 4: The RBs that have multiplied are transformed back into EBs within the infected epithelial cell.
- Phase 5: The infected epithelial cells produced more Chlamydia particles.

The infected cell density in each phase *j* of the developmental process at *t* (time) is represented by $I_j(t), \quad j=1,2,3,4,5$. $\Pi$ is the host body’s rate of producing healthy cells. $\beta$ stands for the contact rate. The rate of the life span of a healthy epithelial cell is $\dfrac{1}{\mu _h}$. $\delta _j\quad j=1,2,3,4$, is the progress rate of the infected cells in phase *j* to phase $j+1$. $\gamma$ is the break-up rate of infected epithelial cells in Phase 5. $N_1$ is then converted back from infectious to EB form. $N_2$ is the remaining noninfectious. $\dfrac{1}{\mu _e}$ and $\dfrac{1}{\mu _r}$ are the rates of life span of infection *E*(*t*) and noninfectious *R*(*t*) Chlamydia body form respectively.

Then, the constructed fractional model is given by the following system of coupled ordinary differential equations:

1 $$\begin{aligned} ^CD^{\alpha }H&=\Pi ^{\alpha }-(\Psi (t)+\mu ^{\alpha }_h)H,\nonumber \\ ^CD^{\alpha } I_1&=\Psi (t)H-\delta ^{\alpha }_1\, I_1,\nonumber \\ ^CD^{\alpha } I_j&=\delta ^{\alpha }_{j-1} I_{j-1}-\delta ^{\alpha }_j I_j,\quad j=2,3,4.\nonumber \\ ^CD^{\alpha } I_5&=\delta ^{\alpha }_4 I_4-\gamma ^{\alpha } I_5,\nonumber \\ ^CD^{\alpha } E&=\gamma ^{\alpha }\,N^{\alpha }_1 I_5-(\beta ^{\alpha } H+\mu ^{\alpha }_e)\, E\nonumber \\ ^CD^{\alpha } R&=\gamma ^{\alpha } N^{\alpha }_2 I_5-\mu ^{\alpha }_r R \end{aligned}$$

Subject to the initial conditions

**Fig. 2:**
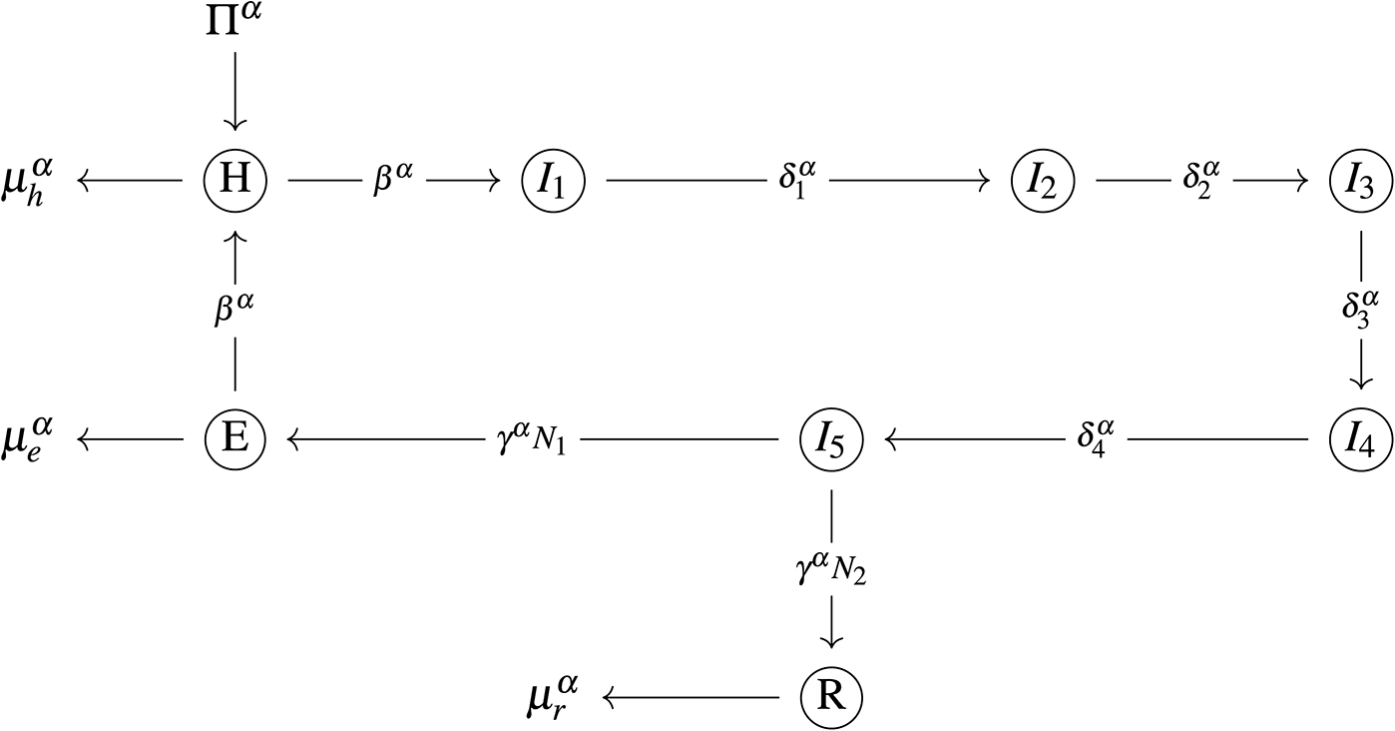
Schematic diagram for the proposed model (1).

$H(0)\ge 0,\,\, I_j(0)\ge 0,\,\, E(0)\ge 0,$  and  $R(0)\ge 0. \quad j=1,2,3,4,5.$   Where $\Psi =\beta ^{\alpha }\,E$. The compartmental links for the proposed model are depicted in Fig. 2.

### Positivity and boundedness

This section shows that the system (1) is biologically well-posed. Moreover, we show that the region $\Omega =\{(H(t), I_j(t), E(t), R(t))\in {\mathbb {R}}^8_+: H(t), I_j(t), E(t), R(t)\le E_{\alpha ,1}(\phi \,t^{\alpha })\}, \quad j=1,\cdots ,5$, is positively invariant, i.e., the solution of system (1) always remains non-negative as long as the initial values are non-negative.

#### Theorem 5

*For* $t\ge 0$, *the non-negative region* $\Omega$ *of solutions is positively invariant for the system* (1).

#### Proof

Let *P*(*t*) be written as the sum of all compartments as follows: $\displaystyle {P(t)= H(t)+\sum \nolimits _{j=1}^5 I_j(t)+E(t)+R(t)},$ therefore:

2 $$\begin{aligned} ^CD^{\alpha } P(t)&= ^CD^{\alpha }H(t)+ ^CD^{\alpha } \sum _{j=1}^5 I_j(t)+ ^CD^{\alpha } E(t)+\, ^CD^{\alpha } R(t)\nonumber \\&\le \phi \, P(t), \quad \text {where}\, \phi \, \text {is the population coefficient.} \end{aligned}$$

To solve for *P*(*t*), we apply the Laplace transform method by taking the Laplace transform for both sides of (2). Assume ${\mathscr {L}}\{P\}(t)={\widehat{P}}(s)$ and by the application of the solvability condition, $P(0)=P_0$, we get

3 $$\begin{aligned} {\mathscr {L}}\{ ^CD^{\alpha } P(t)\}&\le {\mathscr {L}}\{\phi \,P(t)\} \nonumber \\ s^{\alpha }\,{\widehat{P}}(s)-s^{\alpha -1}\,P(0)&\le \phi \, {\widehat{P}}(s)\nonumber \\ {\widehat{P}}(s)&\le \frac{s^{\alpha -1}\,P_0}{s^{\alpha }-\phi }\nonumber \\&= P_0\, \left( \frac{1}{s(1-\frac{\phi }{s^{\alpha }})}\right) \nonumber \\ {\widehat{P}}(s)&= P_0\, \sum _{n=0}^{\infty } \frac{\phi ^n}{s^{n\alpha +1}} \end{aligned}$$

To find *P*(*t*), we take the Laplace inverse of both sides of (3) as follows

$$\begin{aligned} {\mathscr {L}}^{-1}\{ {\widehat{P}}(s)\}&\le {\mathscr {L}}^{-1}\left\{P_0\, \sum _{n=0}^{\infty } \frac{\phi ^n}{s^{n\alpha +1}}\right\} \\ P(t)&\le P_0 \sum _{n=0}^{\infty } \phi ^n {\mathscr {L}}^{-1}\left\{ \frac{1}{s^{n\alpha +1}}\right\} \\&= P_0 \sum _{n=0}^{\infty } \frac{(\phi \,t^{\alpha })^n}{\Gamma (n\alpha +1)}\\ P(t)&= P_0\, E_{\alpha ,1}(\phi \,t^{\alpha }),\quad \text {where}\, E_{\alpha ,1}(\phi \,t^{\alpha })\, \text {is the Mittag-Leffler function}. \end{aligned}$$

Thus, the proof is completed. $\square$

### Existence and uniqueness analysis

In this section, we establish that the solution to the model is both existent and unique (1).

#### Theorem 6

*The kernels of the equations of the proposed system* (1) *satisfy the Lipschitz continuous for* $L_j\ge 0, \quad j=1,\cdots , 8$.

#### Proof

Consider the following steady-state version of (1):

4 $$\begin{aligned} f_1(H)&=\Pi -(\Psi (t)+\mu ^{\alpha }_h)H,\nonumber \\ f_2(I_1)&=\Psi (t)H-\delta ^{\alpha }_1\, I_1,\nonumber \\ f_{j+1}(I_j)&=\delta ^{\alpha }_{j-1} I_{j-1}-\delta ^{\alpha }_j I_j,\quad j=2,3,4.\nonumber \\ f_6(I_5)&=\delta ^{\alpha }_4 I_4-\gamma ^{\alpha } I_5,\nonumber \\ f_7(E)&=\gamma ^{\alpha }\,N^{\alpha }_1\,I_5-(\beta ^{\alpha } H+\mu ^{\alpha }_e)\, E\nonumber \\ f_8(R)&=\gamma ^{\alpha } N^{\alpha }_2 I_5-\mu ^{\alpha }_r R. \end{aligned}$$

Upon applying Theorem (1), we have

$$\begin{aligned} ||f_1(H)-f_1(H^1)||&=||(\Psi (t)+\mu ^{\alpha }_h)(H-H^1)||,\\&\le \left( \max _{t\in [0,\tau ]}||\Psi (t)||+|\mu ^{\alpha }_h|\right) ||H-H^1||,\\&\le L_1 ||H-H^1|| \end{aligned}$$

where

5 $$\begin{aligned} L_1=\max _{t\in [0,\tau ]}||\Psi (t)||+|\mu ^{\alpha }_h| <\infty , \end{aligned}$$

In the same way, we can achieve the following:

$$\begin{aligned} ||f_{j+1}(I_j)-f_{j+1}(I^1_j)||&\le L_{j+1} ||(I_j-I_j^1)||,\quad j=1,2,3,4,5.\\ ||f_7(E)-f_7(E^1)||&\le L_7||E-E^1||,\\ ||f_8(R)-f_8(R^1)||&\le L_8 ||R-R^1||, \end{aligned}$$

where

$L_{j+1}=|\delta _j^{\alpha }|< \infty , \quad j=1,2,3,4.$, $L_6=|\gamma ^{\alpha }|<\infty$, $\displaystyle { L_7=|\beta ^{\alpha }|\,\max _{t\in [0,\tau ]}||H||+|\mu _e^{\alpha }|<\infty }$, and $L_8=|\mu _r^{\alpha }|<\infty$. Which completes the proof. $\square$

#### Lemma 2

The equations in the model (1) can be converted into equivalent Volterra-integral equations.

#### Proof

By considering the system (1)

6 $$\begin{aligned} ^CD^{\alpha } x_j(t)=f_j(t,x_j), \quad j=1,\cdots , 8. \end{aligned}$$

By applying Definition (5) on the two sides of Eq. (6), we conclude:

7 $$\begin{aligned} I^{\alpha } ( ^CD^{\alpha } x_j(t))=I^{\alpha } (f_j(t,x_j)), \quad j=1,\cdots , 8. \end{aligned}$$

The left-hand side becomes $x_j(t)-x_j(0)$ and hence Eq. (7) becomes

8 $$\begin{aligned} x_j(t)-x_j(0)=\frac{1}{\Gamma (\alpha )} \int _0^t (t-\tau )^{\alpha -1} f_j(\tau ,x_j)d\tau , \quad \text {for all}\,\, j. \end{aligned}$$

Therefore, the proof follows, and the equations of the model (1) are transformed to equivalent Volterra-integral equations as follows:

9 $$\begin{aligned} x_j(t)= x_j(0)+\frac{1}{\Gamma (\alpha )} \int _0^t f_j(\tau ,x_j) (t-\tau )^{\alpha -1} \,d\tau , \quad \text {for all} \,\,j. \end{aligned}$$

$$\square$$

#### Theorem 7

^32^ *For* $0<\alpha <1$ *and* $G=[0,\tau ]\subseteq {\mathbb {R}},\, J=[x_j(0)-k,x_j(0)+k]$. *Let* $f_j:G\times J\longrightarrow {\mathbb {R}}$ *be Lipschitz condition and continuous bounded i*.*e*. $\exists ! M_j>0,\, ||f_j(x)||<M_j$, *where* $M_j=\max _{t\in [0,\tau ]} ||f_j(x)||.$ *If* $\dfrac{L_j\,k_j}{M_j}<1$, *then* $\exists$ *only one solution for the initial value problem* (1), *namely*, $x_j\in C[0,\tau ^*]$, *where* $\tau ^*=\min \left\{ \tau ,\left( \dfrac{k_j\Gamma (\alpha +1)}{M_j}\right) ^{\dfrac{1}{\alpha }}\right\}$.   $j=1,2,\cdots , 8$.

#### Proof

Let $X=\{x_j(t)\in C([0,\tau ], {\mathbb {R}}^8): ||x_j(t)-x_j(0)||\le k_j$, $\quad j=1,2,\cdots , 8$. Since every sequence $x_j^n$ in *X* converges to $x_j\in C([o,\tau ],{\mathbb {R}}^8)$ with respect to infinity norm, $||\cdot ||_{\infty }$, and *x* is continuous and $||x_j(t)-x_j(0)||\le k_j$, then $\forall n\, ||x_j^n(t)-x(0)||\le k_j$. Thus, *X* is closed and hence a complete metric space.

Define $F: X\longrightarrow X$ such that

$$\displaystyle {F_j(x(t))=x_j(0)+\frac{1}{\Gamma (\alpha )}\,\int _0^t k_\alpha (\tau ,\,t)\,f_j(\tau ,x_j(t))\,d\tau }$$, where $$k_\alpha (\tau ,\,t) = (t-\tau )^{\alpha -1}$$ then

$$\begin{aligned} ||F_{j}(x_j(t))-x_j(0)||&= \left\Vert \frac{1}{\Gamma (\alpha )}\,\int _0^t k_\alpha (\tau ,\,t)\,f_j(\tau ,x_j(t))\,d\tau \right\Vert ,\quad j=1,2,\cdots ,8.\\&\le \frac{1}{\Gamma (\alpha )}\,\int _0^t k_\alpha (\tau ,\,t)\,||f_j(\tau ,x_j(t))||\,d\tau \\&\le \frac{M_j}{\Gamma (\alpha )}\,\int _0^t k_\alpha (\tau ,\,t)\,d\tau \\&=\frac{M_j\,t^{\alpha }}{\Gamma (\alpha +1)} \le \frac{M_j\,(\tau ^*)^{\alpha }}{\Gamma (\alpha +1)}\le k_j \end{aligned}$$

Therefore, $||F_{j}(x_j(t))-x_j(0)||\le k_j$, and hence, *F* maps *X* onto itself.

To show that *F* is a contraction operator, we assume that $y,z\in X$, such that

$$\begin{aligned} \Vert F_{j}(y_j(t))-F_j(z_j(t))\Vert&= \left\Vert \frac{1}{\Gamma (\alpha )}\,\int _0^t k_\alpha (\tau ,\,t)\,(f_j(\tau ,y_j(t))-f_j(\tau ,z_j(t))\,d\tau \right\Vert ,\quad j=1,2,\cdots ,8.\\&\le \frac{1}{\Gamma (\alpha )}\,\int _0^t k_\alpha (\tau ,\,t)\,||(f_j(\tau ,y_j(t))-f_j(\tau ,z_j(t))||\,d\tau \\&\le \frac{L_j}{\Gamma (\alpha )}\,||y_j(t)-z_j(t)||\,\int _0^t k_\alpha (\tau ,\,t)\,d\tau \\&=\frac{L_j\,t^{\alpha }}{\Gamma (\alpha +1)}\,||y_j(t)-z_j(t)||\\&\le \frac{L_j\,(\tau ^*)^{\alpha }}{\Gamma (\alpha +1)}\le \frac{L_j\,k_j}{M_j}\,||y_j(t)-z_j(t)||. \end{aligned}$$

Therefore, $\Vert F_{j}(y_j(t))-F_j(z_j(t))\Vert \le \frac{L_j\,k_j}{M_j}\,||y_j(t)-z_j(t)||$. Thus, by the assumption $\dfrac{L_j\,k_j}{M_j}<1$, *F* is a contraction mapping. Therefore, it possesses a unique fixed point. Additionally, the system (1) has only one solution. $\square$

## Equilibrium stability analysis

In this section, we calculated the reproduction number, ${\mathscr {R}}_0$. Additionally, we identified the equilibrium points and examined the stability of the proposed model (1).

### Mathematical determination of ${\mathscr {R}}_0$

The basic reproduction number ${\mathscr {R}}_0$ is a fundamental epidemiological measurement used to assess the potential transmission of infectious diseases within a population. It quantifies the mean number of secondary infections caused by an infected individual in a population where every case is susceptible. If $R_0$ is bigger than 1, it indicates that the disease can sustain transmission and is likely to cause an outbreak. Conversely, if $R_0$ is smaller than 1, the disease will ultimately die out as infected individuals cannot pass it on to others. Understanding and estimating $R_0$ is crucial for public health officials and policymakers in implementing effective control measures to contain and manage infectious disease outbreaks. We utilize the advanced matrix theorem to calculate the basic reproduction number, $R_0$^33^. We express the rate of new infections regarding different compartments’ transmission and recovery rates. Then, by using the notation of^34^, we divided the infection states into transmission and transition parts as follows:

$$\begin{aligned} F=\begin{pmatrix} 0& 0& 0& 0& 0& \frac{\beta ^{\alpha }\Pi ^{\alpha }}{\mu _h^{\alpha }}& 0\\ 0& 0& 0& 0& 0& 0& 0\\ 0& 0& 0& 0& 0& 0& 0\\ 0& 0& 0& 0& 0& 0& 0\\ 0& 0& 0& 0& 0& 0& 0\\ 0& 0& 0& 0& 0& 0& 0\\ 0& 0& 0& 0& 0& 0& 0\\ \end{pmatrix}, \text { and}\,\, V=\begin{pmatrix} \delta _1^{\alpha }& 0& 0& 0& 0& 0& 0\\ -\delta _1^{\alpha }& \delta _2^{\alpha }& 0& 0& 0& 0& 0\\ 0& -\delta _2^{\alpha }& \delta _3^{\alpha }& 0& 0& 0& 0\\ 0& 0& -\delta _3^{\alpha }& \delta _4^{\alpha }& 0& 0& 0\\ 0& 0& 0& -\delta _4^{\alpha }& \gamma ^{\alpha }& 0& 0\\ 0& 0& 0& 0& -\gamma ^{\alpha }\,N_1^{\alpha }& \frac{\beta ^{\alpha }\Pi ^{\alpha }}{\mu _h^{\alpha }}+\mu _e^{\alpha }& 0\\ 0& 0& 0& 0& -\gamma ^{\alpha }\,N_2^{\alpha }& 0& \mu _r^{\alpha }\\ \end{pmatrix} \end{aligned}$$

Upon using theorem (2) in^34^, we can find ${\mathscr {R}}_0$ is $\textit{spectral radius}(F\times V^{-1})$. Thus, the basic reproduction is given by the following formula:

10 $$\begin{aligned} {\mathscr {R}}_0=\frac{\beta ^{\alpha }\,\Pi ^{\alpha }\,N_1^{\alpha }}{\beta ^{\alpha }\Pi ^{\alpha }+\mu _h^{\alpha }\,\mu _e^{\alpha }} \end{aligned}$$

### Existence of equilibria

To obtain the model (1) Chlamydia-free equilibrium point (CFE), we set the right-hand side of the system (1) to zero as follows:

11 $$\begin{aligned} \Pi ^{\alpha }-(\Psi (t)+\mu ^{\alpha }_h)H&=0,\nonumber \\ \Psi (t)H-\delta ^{\alpha }_1\, I_1&=0,\nonumber \\ \delta ^{\alpha }_{j-1} I_{j-1}-\delta ^{\alpha }_j I_j&=0,\quad j=2,3,4.\nonumber \\ \delta ^{\alpha }_4 I_4-\gamma ^{\alpha } I_5&=0,\nonumber \\ \gamma ^{\alpha }\,N^{\alpha }_1-(\beta ^{\alpha } H+\mu ^{\alpha }_e)\, E&=0\nonumber \\ \gamma ^{\alpha } N^{\alpha }_2 I_5-\mu ^{\alpha }_r R&=0 \end{aligned}$$

Where $\Psi (t)=\beta ^{\alpha } \,E$. The solution of the system (12) is given by ${\mathscr {E}}_0=\left( \frac{\Pi ^{\alpha }}{\mu _h^{\alpha }}, 0, 0, 0, 0, 0, 0, 0\right) .$

### Local stability

In epidemic models, local stability analysis is important for determining whether equilibrium points are stable and understanding how diseases spread in a population. Insights regarding the system’s long-term behavior and the possibility of disease outbreaks may be gained by examining the stability of these equilibria. The Jacobian matrix of the model system (1) is given as follows:

$$\begin{aligned} J=\begin{pmatrix} -\beta ^{\alpha }\,E-\mu _h^{\alpha }& 0& 0& 0& 0& 0& 0\\ \beta ^{\alpha }\,E& -\delta _1^{\alpha }& 0& 0& 0& 0& 0\\ 0& \delta _1^{\alpha }& -\delta _2^{\alpha }& 0& 0& 0& 0\\ 0& 0& \delta _2^{\alpha }& -\delta _3^{\alpha }& 0& 0& 0\\ 0& 0& 0& \delta _3^{\alpha }& -\delta _4^{\alpha }& 0& 0\\ 0& 0& 0& 0& \delta _4^{\alpha }& -\gamma ^{\alpha }& 0\\ 0& 0& 0& 0& 0& \gamma ^{\alpha }\,N_1^{\alpha }& -\beta ^{\alpha }\, H-\mu _e^{\alpha }\\ \end{pmatrix} \end{aligned}$$

#### Theorem 8

*The model exhibits local asymptotic stability (LAS) at the disease-free equilibrium* ${\mathscr {E}}_0$.

#### Proof

The $J({\mathscr {E}}_0)$ Jacobian matrix at the disease-free equilibrium point, ${\mathscr {E}}_0=\left( \frac{\Pi ^{\alpha }}{\mu _h^{\alpha }}, 0, 0, 0, 0, 0, 0, 0\right)$, is given as follows:

$$\begin{aligned} J(\mathscr {E}_0)=\begin{pmatrix} 0& 0& 0& 0& 0& 0& 0\\ 0& -\delta _1^{\alpha }& 0& 0& 0& 0& 0\\ 0& \delta _1^{\alpha }& -\delta _2^{\alpha }& 0& 0& 0& 0\\ 0& 0& \delta _2^{\alpha }& -\delta _3^{\alpha }& 0& 0& 0\\ 0& 0& 0& \delta _3^{\alpha }& -\delta _4^{\alpha }& 0& 0\\ 0& 0& 0& 0& \delta _4^{\alpha }& -\gamma ^{\alpha }& 0\\ 0& 0& 0& 0& 0& \gamma ^{\alpha }\,N_1^{\alpha }& -\frac{\beta ^{\alpha }\,\Pi ^{\alpha }}{\mu _h^{\alpha }} -\mu _e^{\alpha }\\ \end{pmatrix} \end{aligned}$$

Upon solving the characteristic equation of the Jacobian matrix $J({\mathscr {E}}_0)$, $i.e.,\, |\lambda \,I-J({\mathscr {E}}_0)|=0$, we obtain the following eigenvalues of $J({\mathscr {E}}_0)$:

$$\begin{aligned}\lambda _1=0,\,\lambda _2=-\delta _1^{\alpha }, \, \lambda _3=-\delta _2^{\alpha }, \, \lambda _4=-\delta _3^{\alpha }, \,\lambda _5=-\delta _4^{\alpha },\, \lambda _6=-\gamma ^{\alpha },\, \text {and}\,\, \lambda _7=-\dfrac{\beta ^{\alpha }\,\Pi ^{\alpha }}{\mu _h^{\alpha }} -\mu _e^{\alpha } \end{aligned}$$

Since the eigenvalues of $J({\mathscr {E}}_0)$, $\lambda _2$ to $\lambda _7$ are negative real numbers, then $|\arg (\lambda _j)|=\pi >\dfrac{\alpha \pi }{2},\quad 0<\alpha <1,\quad \forall j=1,\cdots , 7$. Thus, all the nonzero eigenvalues of the Jacobian matrix $J({\mathscr {E}}_0)$ are negative and satisfying $|\arg (\lambda _j)|=\pi >\dfrac{\alpha \pi }{2},\quad 0<\alpha <1,\quad \forall j=1,\cdots , 7$, then by theorem (2), the equilibrium point ${\mathscr {E}}_0$ is locally asymptotically stable. $\square$

## Endemic equilibrium

At endemic equilibrium, infection would have prevailed in a community for a long time but with general stability and persistence. This is because, at this balance, the rate of new infections is already balanced with the rate of recovery or acquisition of immunity; therefore, the prevalence of the disease will remain constant within the population. Most of these endemic diseases are well-established and integral to the region’s general health landscape. Therefore, most public health programs focus on containing and controlling such endemic diseases to prevent outbreaks and reduce the impact on a population’s general health and well-being. The attainment of endemic equilibrium will call for a cocktail of initiatives that includes vaccination, setting up effective healthcare structures, and education and awareness among people.

### Existence of an endemic equilibrium point

Let ${\mathscr {E}}_1^*=(H^*, I_1^*, I_2^*, I_3^*, I_4^*, I_5^*, E^*, R^*)$ be an equilibrium point of the proposed model (1).

#### Theorem 9

*The proposed model* (1) *has a unique endemic equilibrium point, *${\mathscr {E}}_1^*$, *if and only if* ${\mathscr {R}}_0$ *more than 1*.

#### Proof

To determine the existence condition for Chlamydia body forms within the infected host’s body, consider $\Psi ^*=\beta ^{\alpha }\, E^*$ as the effective force of infection in the model (1) at equilibrium. Thus, we assume that ${\mathscr {E}}_1^*$ all coordinates are non-zeros, and hence solving the model (1) at steady state yields;

$$\begin{aligned}H^*=\frac{\Pi ^{\alpha }}{\Psi ^*+\mu _h^{\alpha }}, \quad I_1^*=\frac{\Psi ^*\,H^*}{\delta _1^{\alpha }},\quad I_2^*=\frac{\Psi ^*\,H^*}{\delta _2^{\alpha }},\quad I_3^*=\frac{\Psi ^*\,H^*}{\delta _3^{\alpha }}\quad I_4^*=\frac{\Psi ^*\,H^*}{\delta _4^{\alpha }}, \end{aligned}$$

12 $$\begin{aligned} I_5^*=\frac{\Psi ^*\,H^*}{\gamma ^{\alpha }}, \quad E^*=\frac{\Psi ^*\,H^*\,(N_1^{\alpha }-1)}{\mu _e^{\alpha }},\quad \text {and} \quad R^*=\frac{\Psi ^*\,H^*\,N_2^{\alpha }}{\mu _r^{\alpha }} \end{aligned}$$

Substituting the values in Eq. (12) in $\Psi ^*=\beta ^{\alpha }\,E^*$, we get: $\Psi ^*=\dfrac{\beta ^{\alpha }\,\Psi ^*\,\Pi ^{\alpha }\,(N_1^{\alpha }-1)}{\mu _e^{\alpha }\,(\Psi ^*+\mu _h^{\alpha })}$ which can be simplified to

$$\begin{aligned} \Psi ^*=\frac{(\beta ^{\alpha }\,\Pi ^{\alpha }+\mu _e^{\alpha }\,\mu _h^{\alpha })}{\mu _e^{\alpha }}\,\left[ {\mathscr {R}}_0-1\right] \end{aligned}$$

Thus, if ${\mathscr {R}}_0<1$, then $\Psi ^*<0$ and hence the system has no biological meaning. Therefore, by the positivity of $\Psi ^*$, the endemic equilibrium point, ${\mathscr {E}}_1^*$, exists whenever the reproduction number ${\mathscr {R}}_0$ is greater than one. $\square$

### Global stability analysis

In this section, the global stability analysis of the Chlamydia present (endemic) equilibrium point is conducted, and the following result is obtained.

#### Theorem 10

*The Chlamydia present (endemic) equilibrium point*, ${\mathscr {E}}_1^*$, *is globally asymptotically stable if* ${\mathscr {R}}_0>1$

#### Proof

Let ${\mathscr {R}}_0>1$, then by theorem (9), model (1) has only one Chlamydia-present equilibrium point ${\mathscr {E}}_1^*$. Define the following Lyapunov function:

13 $$\begin{aligned} {\mathscr {F}}(H,I_1,I_2,I_3,I_4,I_5,E)=\left[ H-H^*-H^*\,\ln {\frac{H}{H^*}}\right] +\sum _{j=1}^5\left[ I_j-I^*_j-I^*_j\,\ln {\frac{I_j}{I^*_j}}\right] +\left[ E-E^*-E^*\,\ln {\frac{E}{E^*}}\right] \end{aligned}$$

Take Caputo $\alpha -$derivative of both sides for the Lyapunov operator, ${\mathscr {F}}$, in Eq. (13), and by the application of the linear property (4), we have

14 $$\begin{aligned} ^CD^{\alpha }\,{\mathscr {F}}=^CD^{\alpha }\,\left[ H-H^*-H^*\,\ln {\frac{H}{H^*}}\right] +^CD^{\alpha }\,\sum _{j=1}^5\left[ I_j-I^*_j-I^*_j\,\ln {\frac{I_j}{I^*_j}}\right] +^CD^{\alpha }\,\left[ E-E^*-E^*\,\ln {\frac{E}{E^*}}\right] \end{aligned}$$

By the application of theorem (3) on Eq. (14), we get:

15 $$\begin{aligned} ^CD^{\alpha }\,{\mathscr {F}}\le \left[ 1-\frac{H^*}{H}\right] \, ^CD^{\alpha }\,H +\sum _{j=1}^5\left[ 1-\frac{I^*_j}{I_j}\right] \, ^CD^{\alpha }\,I_j+\left[ 1-\frac{E^*}{E}\right] \, ^CD^{\alpha }\,E \end{aligned}$$

Substituting the values of the Caputo derivatives from the system (1) into Eq. (15), we obtain:

$$\begin{aligned} ^CD^{\alpha }\,{\mathscr {F}}&\le \beta ^{\alpha }\,E^*\,H^*\left[ 7-\frac{H^*}{H}-\frac{I_5}{I_5^*}-\frac{H\,E\,I_1^*}{H^*\,E^*\,I_1}-\frac{I_1\,I_2^*}{I_1^*\,I_2}-\frac{I_2\,I_3^*}{I_2^*\,I_3}-\frac{I_3\,I_4^*}{I_3^*\,I_4}-\frac{I_4\,I_5^*}{I_4^*\,I_5}\right] \\&\quad + \mu _h^{\alpha }\,H^*\left[ 2-\frac{H}{H^*}-\frac{H^*}{H}\right] \\&\quad -\beta ^{\alpha }\,\left( E(H-\mu _e^{\alpha })+N_1^{\alpha }\,H^*\,E^*\,\frac{I_5}{I_5^*}\right) \left[ 1-\frac{E^*}{E}\right] \end{aligned}$$

By the conclusion of the Arithmetic-Geometric Mean inequality (AGM), $\dfrac{a+b}{2}\le a\,b$, we let $a=\dfrac{H}{H^*}$ and $b=\dfrac{H^*}{H}$ to get that

16 $$\begin{aligned} \left[ 2-\frac{H}{H^*}-\frac{H^*}{H}\right]&= 2-(a+b)\nonumber \\&= 2\left( 1-\frac{a+b}{2}\right) \quad \text {by the (AGM) inequality}\nonumber \\&\le 2(1-ab), \quad \text {but}\,\, ab=1\nonumber \\&=0 \end{aligned}$$

Which concludes that $\left[ 2-\frac{H}{H^*}-\frac{H^*}{H}\right] \le 0$. Upon applying the same argumentation, we can conclude that

$$\begin{aligned} \left[ 7-\frac{H^*}{H}-\frac{I_5}{I_5^*}-\frac{H\,E\,I_1^*}{H^*\,E^*\,I_1}-\frac{I_1\,I_2^*}{I_1^*\,I_2}-\frac{I_2\,I_3^*}{I_2^*\,I_3}-\frac{I_3\,I_4^*}{I_3^*\,I_4}-\frac{I_4\,I_5^*}{I_4^*\,I_5}\right] \le 0. \end{aligned}$$

But, $$\beta ^{\alpha }\,\left( E(H-\mu _e^{\alpha })+N_1^{\alpha }\,H^*\,E^*\,\frac{I_5}{I_5^*}\right) \left[ 1-\frac{E^*}{E}\right] \ge 0$$.

Therefore, we conclude that $^CD^{\alpha }\,{\mathscr {F}}\le 0$, and by theorem (4), the Chlamydia-present equilibrium point ${\mathscr {E}}_1^*$ is globally asymptotically stable. $\square$

## Discussion and conclusion

### Sensitivity analysis

In light of the uncertainty surrounding the value of the parameters, we conducted an analysis using a variety of parameters to understand how changes in these parameters affect the basic reproduction number ($R_0$) within our model. We used Latin Hypercube Sampling and Partial Rank Correlation Coefficients (PRCCs) for this analysis to identify the parameters that significantly impact $R_0$^35–37^. Latin Hypercube Sampling is used in statistical sampling to evaluate the sensitivity of a result variable to all input variables. On the other hand, PRCCs are used to measure the relative sensitivity of each given parameter, regardless of whether its effect on the result variable is positive or negative. The values for the range of parameters were derived from Table 1 below.

**Table 1:** The characterization of the variables and parameters in the proposed model (1).

Variable	Description	
*H*	The concentration of epithelial cells that are in a healthy state.	
$I_j$	The concentration of epithelial cells that are infected in phase $j=1,\,2,\,3,\,4,\,5$.	
*E*	Concentration of Chlamydia in the Elementary Body (EB) form.	
*R*	Concentration of Chlamydia in the Reticulate Body (RB) form.	

Parameter	Description	Sample value utilized
$N_1^\alpha$	Quantity of EB forms generated per bursting cell	Assumed to range between 150 and 400
$N_2^\alpha$	Quantity of RB forms generated per bursting cell	Assumed to range between 50 and 100
$\beta ^\alpha$	Contact rate between host epithelial cells and EB forms	Assumed range: [0.04, 0.1]
$\mu _h$	Average lifespan of healthy epithelial cells	Assumed as $\frac{1}{10}$
$\mu _e$	Average lifespan of Chlamydia EB forms	Assumed as $\frac{1}{10}$
$\mu _r$	Average lifespan of Chlamydia RB forms	Assumed as $\frac{1}{6}$
$\delta _1^\alpha$	Rate of progression of infected cells from phase 1 to phase 2	Taken from References^31,38^: $\frac{1}{8}$
$\delta _2^\alpha$	Rate of progression of infected cells from phase 2 to phase 3	Taken from References^31,38^: $\frac{1}{4}$
$\delta _3^\alpha$	Rate of progression of infected cells from phase 3 to phase 4	Taken from References^31,38^: $\frac{1}{18}$
$\delta _4^\alpha$	Rate of progression of infected cells from phase 4 to phase 5	Taken from References^31,38^: $\frac{1}{10}$
$\gamma ^{\alpha }$	Rate of disintegration of infected cells in phase 5	Taken from References^31,38^: $\frac{1}{24}$
$\Pi$	Production rate of healthy epithelial cells	Assumed as 1

**Fig. 3:**
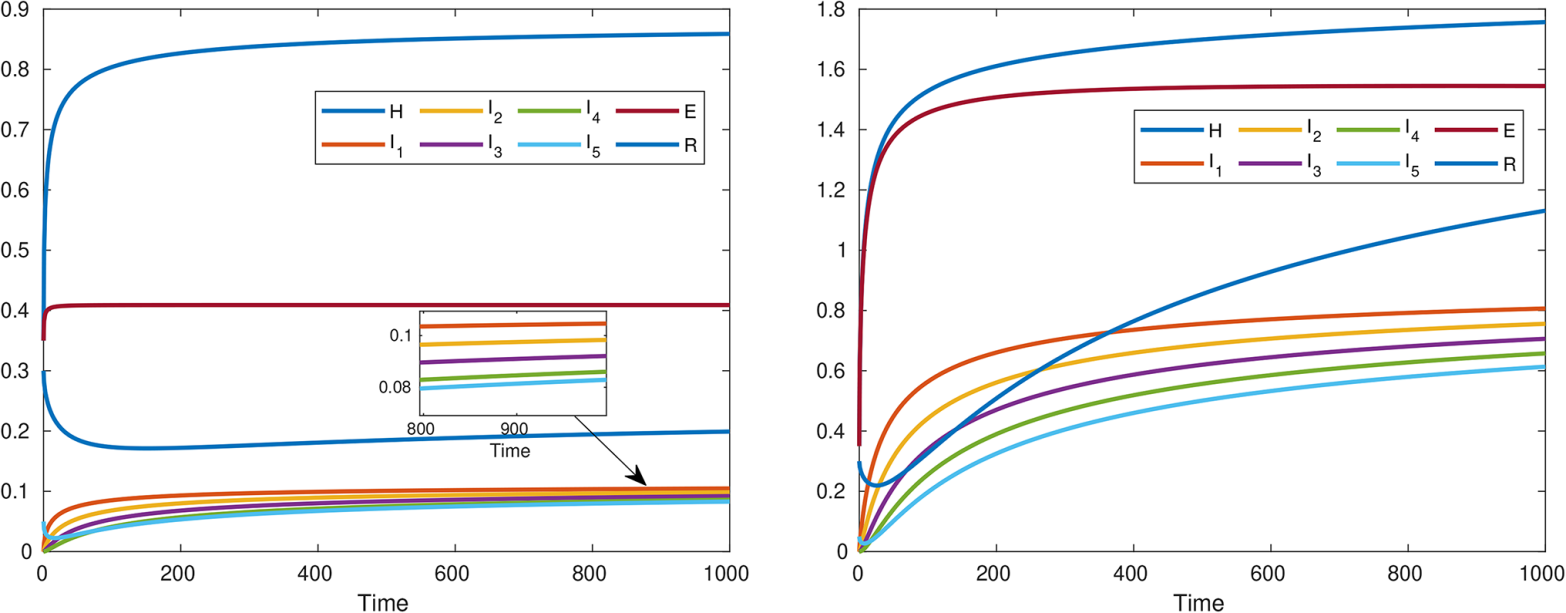
Endemic scenario (left subfigure). Pandemic scenario (right subfigure).

The left sub-figure 3 portrays an endemic situation. Here, Chlamydia persists in a steady state within the population. It’s like a long-standing dance between the bacteria and the host cells. The infection rate remains relatively stable, and the memory of past infections lingers. Imagine a graph where the number of infected individuals fluctuates around a certain equilibrium point. Here, the fractional derivatives step in, allowing us to grasp the more subtle nuances of this persistent equilibrium. The right sub-figure 3 is the realm of chaos and rapid spread! Imagine Chlamydia cases suddenly shooting through the roof like wildfire through a dry forest. In that case, the fractional derivatives account for the memory effects, whereby the system remembers its past states during this pandemic and produces exponential growth. In modeling this memory-driven acceleration, these terms of fractional order turn out to be helpful.

**Fig. 4:**
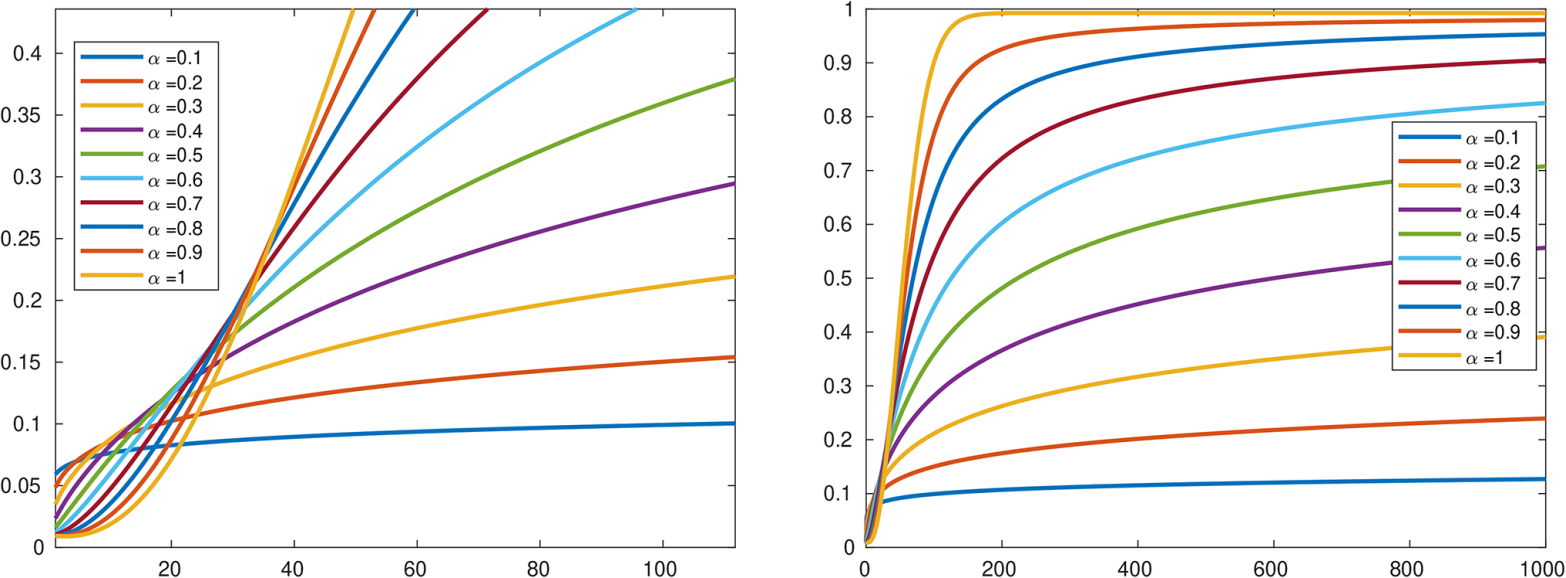
Chlamydia spread dynamics as a function of the fractional order. Along the time, we vary the fractional order from low to high values.

The behavior Across Fractional Orders in Fig. 4 shows at lower fractional orders (close to integer values), the graph shows an endemic equilibrium. Chlamydia persists in a steady state within the population. The infection rate remains relatively stable, akin to a long-standing dance between the bacteria and host cells. As we increase the fractional order, the graph exhibits persistent behavior. Chlamydia becomes a long-term resident, neither fading away nor exploding into a pandemic. This persistence arises from the system’s memory effects-it remembers past states and adjusts its strategy accordingly. Beyond a certain threshold, the graph sharply rises. Chlamydia becomes an accelerated sprinter, rapidly infecting the population. These scenarios often correspond to pandemic situations. The system’s memory accelerates the spread, leading to explosive growth.

**Fig. 5:**
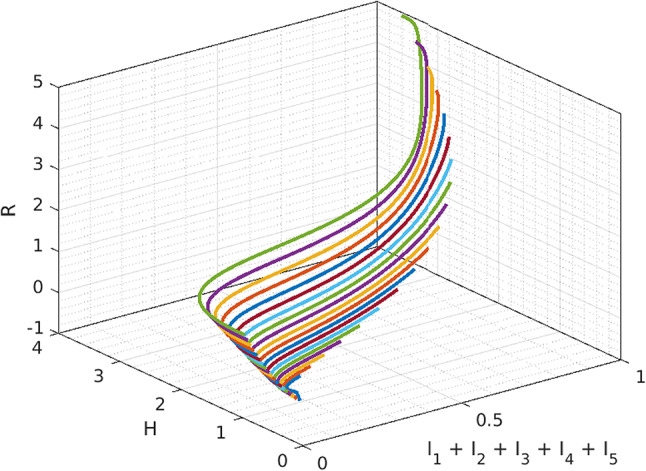
Global stability description.

In Fig. 5, We investigate the system’s long-term behavior by analyzing its equilibrium points and stability. The initial conditions determine whether the system converges to an endemic equilibrium or exhibits oscillations. By varying the initial values of $H,\, R$ and $I_1+I_2+I_3+I_4+I_5$ we explore the system’s global stability.

**Fig. 6:**
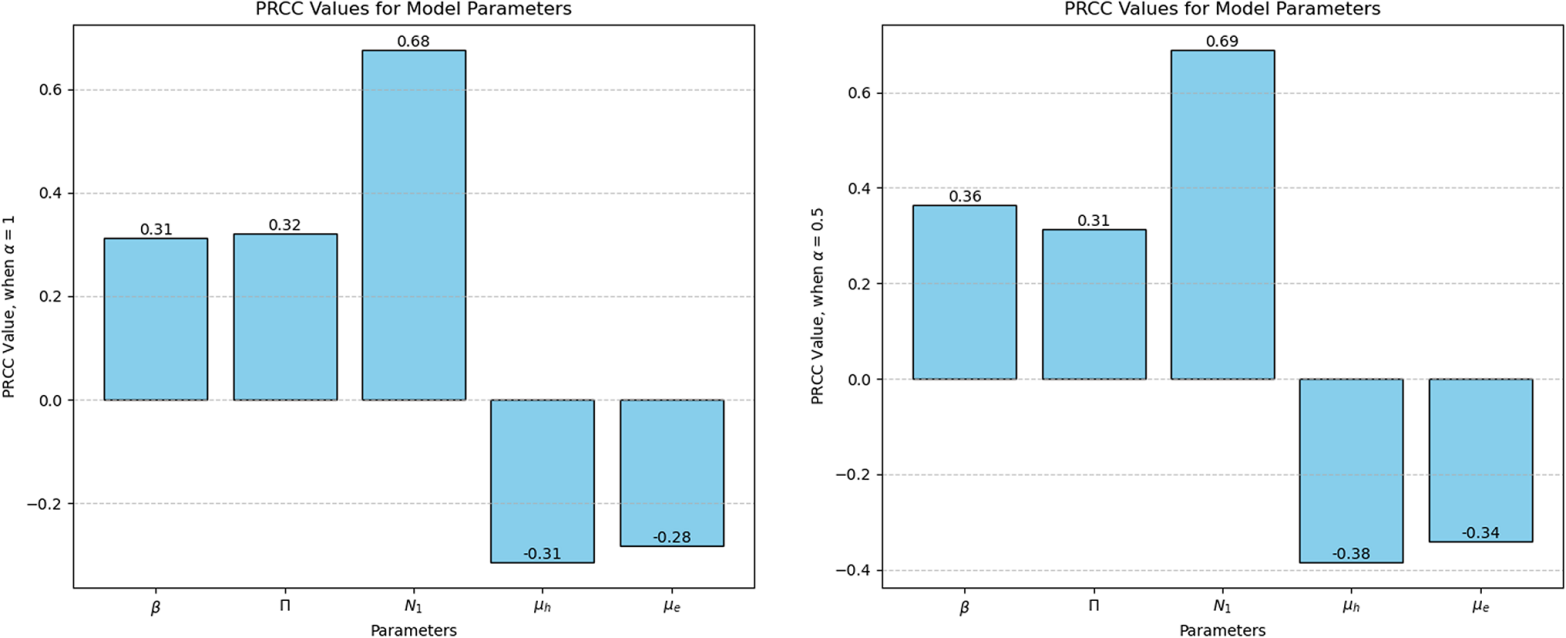
Partial Rank Correlation Coefficient (PRCC) values for model parameters and the reproduction number ($R_0$). The left subfigure shows results for the classical derivative ($\alpha = 1$), while the right subfigure presents findings for the fractional derivative ($\alpha = 0.5$).

Figure 6 describes the PRCC values; these values indicate how changes in each parameter impact the overall Chlamydia spread. A higher PRCC value implies greater sensitivity.

**Fig. 7:**
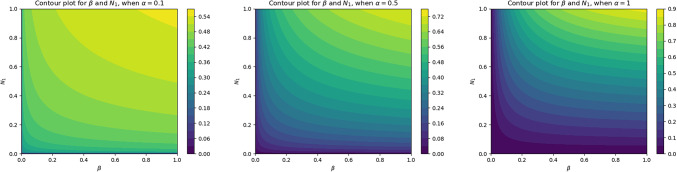
The contour plot represents the relationship between two key parameters $(\beta )$ and $(N_1)$ for Chlamydia spreading.

The contour plots in Fig. 7 illustrate how the basic reproduction number $(R_0)$ for Chlamydia varies with changes in the transmission rate $(\beta )$ and a parameter $(N_1)$. The plots show regions with increasing $(R_0)$ values correspond to higher transmission rates and potential outbreaks. The contour lines connect points with equal $(R_0)$ values, helping to visualize how different combinations of $(\beta )$ and $(N_1)$ influence the transmission potential of Chlamydia.

Using fractional derivatives in these models introduces a memory effect, meaning past interactions within the host population influence current and future disease dynamics. This memory-driven acceleration can increase $(R_0)$ values as the disease ’remembers’ its past encounters and spreads more effectively. Understanding these relationships is crucial for predicting disease dynamics and implementing effective control measures.

A comparison of the proposed model with other models based on the following features called Key Comparative Indicators (KCI) is presented in Table 2.

- Number of Compartments ($KCI_1$): Increasing the number of compartments in epidemiological models can significantly improve the understanding and prediction of disease spread.
- Memory Effects ($KCI_2$): Fractional derivatives account for the history of the process, meaning they can model systems where the current state depends on all past states, not just the immediate past. This is particularly useful in epidemiology, where long-term factors can influence the spread of a disease.
- Sensitivity Analysis of $R_0$ ($KCI_3$): Involves examining how changes in model parameters affect $R_0$.
- Vaccination ($KCI_4$): Vaccination directly reduces the number of susceptible individuals in the population. By immunizing a portion of the population, the overall pool of individuals who can contract and spread Chlamydia is decreased.
- Prediction Accuracy ($KCI_5$): Accurate prediction in each compartment of a Chlamydia model is crucial for understanding the dynamics of the disease and implementing effective control measures.
- Stages of the Developmental Cycle and Density of Infected Cells in Stage ($KCI_6$): Refers to tracking the number of cells infected with Chlamydia at various stages of its unique biphasic life cycle, providing insights into disease progression and treatment efficacy.

**Table 2:** Comparison of epidemiological models for Chlamydia and Gonorrhea.

Model	$KCI_1$	$KCI_2$	$KCI_3$	$KCI_4$	$KCI_5$	$KCI_6$
$S E I_A I_S R$^39^	4	✗	✗	✗	✓	✗
*SVEITRN*^40^	5	✓	✗	✓	✓	✗
$S_H V_{CL} I_{CL} T_{CL} I_G T_G I_{GCL}$^41^	7	✓	✗	✓	✓	✓
$S E I_A I_S$^42^	4	✗	✓	✓	✓	✗
$H_e I_1 I_2 I_3 I_4 I_5 E_b R_b$^29^	8	✗	✓	✓	✓	✓
Proposed Model	8	✓	✓	✓	✓	✓

## Conclusion

In this study, we explored the dynamics of Chlamydia transmission using a fractional order compartmental model within the Caputo framework. The model, validated as biologically accurate, incorporates six categories and provides a positive, bounded, unique solution. We determined the basic reproduction number $(R_0)$, establishing the local stability of the Disease-Free Equilibrium (DFE) and the global stability of the Endemic Equilibrium (EE).

Sensitivity analysis identified key parameters influencing $R_0$: the amount of EB forms generated per bursting cell $(N_1)$, the contact effectiveness rate between host epithelial cells and EB form $(\beta )$, and the average lifespan of healthy epithelial cells $(\mu _h)$. Even minor changes in these parameters significantly affect $R_0$, highlighting their critical role in disease control. Simulations showed that memory effects, represented by the fractional order $(\alpha )$, significantly impact disease spread, with memory-driven acceleration increasing transmission potential. This fractional order model enhances our understanding of Chlamydia spread, offering valuable insights for combating infectious diseases.

## Data Availability

The datasets used and/or analyzed during the current study are available from the corresponding author on reasonable request.
